# Positive association of Parkinson’s disease with ankylosing spondylitis: a nationwide population-based study

**DOI:** 10.1186/s12967-020-02629-w

**Published:** 2020-11-30

**Authors:** Fu-Chiang Yeh, Hsiang-Cheng Chen, Yu-Ching Chou, Cheng-Li Lin, Chia-Hung Kao, Hsin-Yi Lo, Feng-Cheng Liu, Tse-Yen Yang

**Affiliations:** 1 Division of Rheumatology, Immunology, and Allergy, Department of Internal Medicine, Tri-Service General Hospital, National Defense Medical Center, Taipei, Taiwan; 2 Department of Health Promotion and Health Education, National Defense Medical Center, Taipei, Taiwan; 3 School of Medicine, China Medical University, Taichung, Taiwan; 4 Management Office for Health Data, China Medical University Hospital, Taichung, Taiwan; 5 Graduate Institute of Biomedical Sciences, School of Medicine, College of Medicine, China Medical University, Taichung, Taiwan; 6 Department of Nuclear Medicine and PET Center, China Medical University Hospital, Taichung, Taiwan; 7 Department of Bioinformatics and Medical Engineering, Asia University, Taichung, Taiwan; 8 Center of Augmented Intelligence in Healthcare, China Medical University Hospital, Taichung, Taiwan; 9 Graduate Institute of Chinese Medicine, China Medical University, Taichung, Taiwan; 10 Molecular and Genomic Epidemiology Center and Department of Medical Research, China Medical University Hospital, Taichung, Taiwan; 11 Center for General Education & Master Program of Digital Health Innovation, College of Humanities and Sciences, China Medical University, Taichung, Taiwan; 12 Department of Medical Laboratory and Biotechnology, Asia University, Taichung, Taiwan

**Keywords:** Ankylosing spondylitis (AS), Parkinson’s disease (PD), Immunology, National health insurance research database (NHIRD), Retrospective cohort

## Abstract

**Background:**

Ankylosing spondylitis (AS) is characterized by excessive production of inflammatory cytokines. Recent evidence suggests that inflammation underlies the neurodegenerative process of Parkinson’s disease (PD). Whether AS has an influence on the development of PD is unclear. We aimed to examine a relationship, if any exists between AS and PD.

**Methods:**

A population-based matched cohort study was performed using data from the 2000–2010 Taiwan National Health Insurance database. 6440 patients with AS and 25,760 randomly selected, age- and sex-matched controls were included in this study. The risk of PD in the AS cohort was evaluated by using a Cox model.

**Results:**

This study revealed a positive association between AS and the risk of PD regardless of sex and age (aHR 1.75, *p* < .001). Particularly, AS cohort to non-AS cohort relative risk of PD significantly increased for the patients aged below 49 and above 65 years (aHR 4.70, *p* < .001; aHR 1.69, *p* < .001, respectively) and the patients with and without comorbidities (aHR 1.61, *p* < .001; aHR 2.71, *p* < .001, respectively). Furthermore, NSAID use was associated with lower risk of PD (aHR 0.69, *p* < .05). However, the risk of PD was higher (aHR 2.40, *p* < .01) in patients with AS receiving immunosuppressants than in those not receiving (aHR 1.70, *p* < .001).

**Conclusions:**

Patients with AS had an increased risk of PD which might be related to underlying chronic inflammation. Further research is required to elucidate the underlying mechanism.

## Background

Ankylosing spondylitis (AS) is an autoimmune, chronic inflammatory rheumatic disorder that mainly affects the axial skeleton, peripheral joints and entheses, causing severe chronic pain [1]. The development of AS is strongly associated with increased production of tumor necrosis factor-ɑ (TNF-ɑ), interleukin-6 (IL-6) and interleukin-17 (IL-17) [2, 3]. Extra-articular manifestations of AS caused by chronic inflammation, including uveitis, inflammatory bowel disease, osteoporosis, glomerulonephritis and cardiopulmonary involvement, vary widely in terms of frequency and severity [4]. Studies have shown that AS is associated with increased risk of depression, dementia and ischemic stroke; [5–7] however, the underlying mechanism of central nervous system (CNS) involvement in AS is still unknown.

Parkinson’s disease (PD), a common neurodegenerative disorder of the CNS causing bradykinesia, resting tremor, postural instability and muscle rigidity, is pathologically due to progressive cell death of dopaminergic neurons located in the substantia nigra pars compacta [8]. The etiology of PD is multifactorial, however, there is an increasing emphasis that inflammation underlies the neurodegenerative process. Dysfunction of the immune system, such as autoimmune response, may involve in the pathogenesis of the disease. [9] Pro-inflammatory cytokines, specifically TNF-ɑ and IL-6, are significantly increased in the serum and cerebrospinal fluid of PD patients [10–15]. Furthermore, recent studies revealed that serum IL-6 and IL-17 positively correlate with severity of symptoms in PD [12, 14]. The relationship between PD and autoimmune diseases that chronically produce high concentrations of inflammatory cytokines has been reported [16, 17]. However, whether AS is associated with the generation of PD is yet unclear.

In current study, we hypothesized that AS patients are at higher risk of developing PD and we aimed to determine the risk of PD in patients with AS and to identify the associated risk factors by conducting a nationwide longitudinal population-based matched cohort study.

## Methods

### Data Source

This nationwide cohort study was performed by using data from Taiwan’s Longitudinal Health Insurance Database 2000 (LHID2000). The LHID2000 includes all claims data of a million individuals randomly sampled from the 2000 registry for beneficiaries of the National Health Insurance Research Database (NHIRD) [18]. The Taiwanese government established the National Health Insurance (NHI) program in 1995. This single-payer health insurance program enrolled more than 99% of the entire population of 23.74 million people in Taiwan (http://www.nhi.gov.tw/english/index.aspx). Details of the NHI program are described in previous studies [19, 20]. According to the Personal Information Protection Act, the records of patients were de-identified and all researchers signed their agreement for not obtaining privacy of the patients. This study was approved by the Institutional Review Board of China Medical University (CMUH-104-REC2-115).

### Study population

The ankylosing spondylitis (AS) cohort included newly diagnosed AS (ICD-9-CM code 720) patients from 2000 to 2010. The index date for each case was defined as the date of AS diagnosis. According to the age (5-year span), sex, and index year, four non-AS controls were randomly selected from the LHID2000 and frequency-matched to each AS patient. The index date for a non-AS control was assigned as the same as the matched AS patient. Patients with history of PD (ICD-9-CM code 332) before index date were excluded from both cohorts (Table 1).

**Table 1:** Demographic characteristics and co-morbidity in patients with and without ankylosing spondylitis

Variables	Ankylosing spondylitis				*p* value
	No (N = 25,760)		Yes (N = 6440)		
	n	%	n	%	
Sex					0.99
Male	12,396	48.1	3099	48.1	
Female	13,364	51.9	3341	51.9	
Age, years					0.99
20–34	6976	27.1	1744	27.1	
35–49	7588	29.5	1897	29.5	
50–64	6592	25.6	1648	25.6	
≥ 65	4604	17.9	1151	17.9	
Mean (SD) ^†^	47.3	17.1	47.6	16.9	0.15
Comorbidity					
Diabetes	1734	6.73	497	7.72	0.01
Hypertension	6032	23.4	1874	29.1	< 0.001
Hyperlipidemia	3857	15.0	1348	20.9	< 0.001
Stroke	661	2.57	167	2.59	0.90
Depression	868	3.37	375	5.82	< 0.001
Coronary artery disease	2869	11.1	1054	16.4	< 0.001
Head injury	599	2.33	209	3.25	< 0.001
Chronic kidney disease	1368	5.31	532	8.26	< 0.001
Epilepsy	164	0.64	43	0.67	0.78
Medication					
NSAIDs	6053	23.5	1591	24.7	0.04
Immunosuppressant therapy			1282	19.9	
Biological therapy			37	0.57	

Case group mean follow-up 6.72(SD = 3.29)

Control group mean follow-up 6.63(SD = 3.31)

Chi-square test; ^†^Two sample t-test

### Outcome, comorbidity and medication

Both cohorts were followed up from the index date until the PD onset date, December 31, 2011 or withdrawal from the NHI program, whichever came first. Age of the patient was divided into 5-year categories, and the groups were merged when necessary. Baseline comorbidities including diabetes (ICD-9-CM code 250), hyperlipidemia (ICD-9-CM code 272), hypertension (ICD-9-CM codes 401 to 405), stroke (ICD-9-CM codes 430 to 438), depression (ICD-9-CM codes 296.2, 296.3, 300.4, 311), head injury (ICD-9-CM codes 850 to 854, 959.01), coronary artery disease (CAD) (ICD-9-CM codes 410 to 414), chronic kidney disease (ICD-9-CM codes 580 to 589) and epilepsy (ICD-9-CM code 345) considered as risk factors of PD were also analyzed [21]. In addition, the use of nonsteroidal anti-inflammatory drugs (NSAIDs), immunosuppressants (sulfalsalazine and methotrexate) and various biological agents (including adalimumab, golimumab, and etanercept) were analyzed between the two cohorts.

### Statistical analysis

**Fig. 1:**
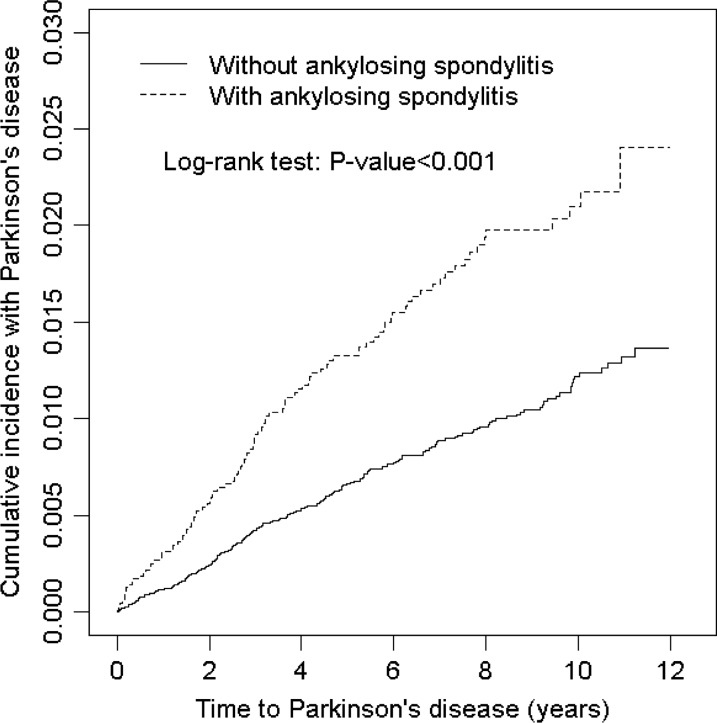
Comparison of cumulative incidence of Parkinson disease between patients with and without ankylosing spondylitis. The Kaplan–Meier plot showed that the AS cohort had a higher cumulative incidence of PD than the non-AS cohort did (log-rank test, *p* < .001)

The chi-square test was used to compare descriptive statistics on demographic status, baseline comorbidities and medication between AS and non-AS cohorts. Student *t* test was used to compare the AS and non-AS cohorts for continuous variables. The Kaplan–Meier method was used to estimate the cumulative incidence of PD in the AS and non-AS cohorts, and the difference was tested using a log-rank test (Fig. 1). The incidence density rates (per 1000 person-years) for PD were calculated. Univariate and multivariate Cox proportional hazards regression analyses were used to calculate the hazard ratios (HRs) with stratification according to sex, age, and comorbidity. The multivariate Cox models were simultaneously adjusted for age and comorbidities of diabetes, hypertension, hyperlipidemia, stroke, depression, coronary artery disease, head injury and chronic kidney disease and medication of NSAIDs. Furthermore, we evaluated the treatment effect of immunosuppressants and biological agents on the risk of Parkinson's disease in the AS cohort. All statistical analyses were conducted using SAS 9.4 software (SAS Institute, Cary, NC, USA). A two-tailed *p* value < 0.05 was considered statistically significant.

## Results

This study included 6440 AS patients and 25,760 non-AS patients with similar age and sex distributions (Table 1). In both cohorts, 51.9% patients were women. The mean age of the patients was approximately 47 years old and 56.6% of the patients were younger than 49 years of age. Comorbidities of diabetes, hypertension, hyperlipidemia, depression, coronary artery disease, head injury, chronic kidney disease, and use of NSAIDs were more prevalent in the AS cohort than in the non-AS cohort (all *p* < 0.05). The mean follow-up period was 6.72 years in the AS cohort and 6.63 years in the non-AS cohort. The Kaplan–Meier analysis showed that the cumulative incidence of Parkinson’s disease was significantly higher in the AS cohort than in the non-AS cohort (log-rank test, *p* < 0.0001) (Fig. 1).

The overall incidence of PD was greater in the AS cohort than in the non-AS cohort with the incidence rates being 2.36 and 1.23 per 1000 person-years, respectively, yielding a crude HR of 1.92 (95% CI 1.52–2.43, *p* < 0.001) and an aHR of 1.75 (95% CI 1.38–2.22, *p* < 0.001) (Table 2). Compared with patients younger than 49 years old, elder patients had higher risk of developing PD (50–64 years, aHR 5.16; 95% CI 3.23–8.23, *p* < 0.001; ≥ 65 years, aHR 21.2; 95% CI 13.5–33.3, *p* < 0.001). The multivariable analysis also demonstrated that PD was independently associated with comorbidities such as hypertension (aHR 1.39; 95% CI 1.05–1.84, *p* < 0.05), stroke (aHR 1.68; 95% CI 1.16–2.43, *p* < 0.01), and depression (aHR 1.97; 95% CI 1.36–2.85, *p* < 0.001). In contrast, the risk of PD was lower in patients taking NSAIDs compared with patients not taking (aHR 0.69; 95% CI 0.50–0.96, *p* < 0.05).

**Table 2:** Incidence and Hazard ratio for Parkinson's disease and Parkinson's disease-associated risk factor

Variable	Event	PY	Rate^#^	Crude HR(95% CI)	Adjusted HR^†^ (95% CI)
Ankylosing spondylitis					
No	210	170,858	1.23	1.00	1.00
Yes	102	43,252	2.36	1.92 (1.52, 2.43)***	1.75 (1.38, 2.22)***
Age, year					
≤ 49	25	125,771	0.20	1.00	1.00
50–64	71	55,426	1.28	6.44 (4.08, 10.2)***	5.16 (3.23, 8.23)***
≥ 65	216	32,913	6.56	32.7 (21.6, 49.5)***	21.2 (13.5, 33.3)***
Sex					
Female	163	103,378	1.58	1.00	1.00
Male	149	110,732	1.35	0.85 (0.68, 1.06)	–
Comorbidity					
Diabetes					
No	269	201,713	1.33	1.00	1.00
Yes	43	12,397	3.47	2.55 (1.85, 3.52)***	0.87 (0.62,1 .21)
Hypertension					
No	111	165,512	0.67	1.00	1.00
Yes	201	48,597	4.14	6.12 (4.85, 7.72)***	1.39 (1.05, 1.84)*
Hyperlipidemia					
No	204	181,940	1.12	1.00	1.00
Yes	108	32,169	3.36	2.97 (2.35, 3.75)***	1.07 (0.83, 1.38)
Stroke					
No	277	210,265	1.32	1.00	1.00
Yes	35	3845	9.10	6.73 (4.73, 9.57)***	1.68 (1.16, 2.43)**
Depression					
No	279	207,215	1.35	1.00	1.00
Yes	33	6895	4.79	3.48 (2.43, 5.00)***	1.97 (1.36, 2.85)***
Coronary artery disease					
No	190	190,490	1.00	1.00	1.00
Yes	122	23,620	5.17	5.13 (4.09, 6.45)***	1.28 (0.99, 1.65)
Head injury					
No	298	209,730	1.42	1.00	1.00
Yes	14	4379	3.20	2.20 (1.29, 3.76)**	1.28 (0.74, 2.20)
Chronic kidney disease					
No	264	202,493	1.30	1.00	1.00
Yes	48	11,617	4.13	3.15 (2.32, 4.28)***	1.11 (0.81, 1.53)
Epilepsy					
No	309	212,960	1.45	1.00	1.00
Yes	3	1150	2.61	1.76 (0.57, 5.50)	–
Medication					
NSAIDs					
No	266	149,655	1.78	1.00	1.00
Yes	46	64,455	0.71	0.41 (0.30, 0.56)***	0.69 (0.50,0 .96)*

Rate^#^, incidence rate, per 1,000 person-years; Crude HR, relative hazard ratio; Adjusted HR^†^: multivariable analysis including age and comorbidities of diabetes, hypertension, hyperlipidemia, stroke, depression, coronary artery disease, head injury and chronic kidney disease and medication of NSAIDs

*p < 0.05, **p < 0.01, ***p < 0.001

The sex-specific AS cohort to non-AS cohort relative risk of PD was significantly higher for both women (aHR 1.66; 95% CI 1.19–2.32, *p* < 0.001) and men (aHR 1.85; 95% CI 1.31–2.62, *p* < 0.01) (Table 3). The PD incidence increased with age, however, the age-specific AS cohort to non-AS cohort relative risk of PD was the highest for the group aged below 49 years (aHR 4.70; 95% CI 2.10–10.5, *p* < 0.001) followed by the group aged above 65 years (aHR 1.69; 95% CI 1.26–2.26, *p* < 0.001). In addition, the comorbidity-specific AS cohort to non-AS cohort relative risk of PD was significant higher both for patients with comorbidity (aHR 1.61; 95% CI 1.23–2.10, *p* < 0.001) and without comorbidity (aHR 2.71; 95% CI 1.63–4.50, *p* < 0.001).

**Table 3:** Incidence rate ratio and hazard ratio of Parkinson's disease and ankylosing spondylitis cohort to non-ankylosing spondylitis cohort

Variables	Ankylosing spondylitis						Crude HR (95%CI)	Adjusted HR^†^ (95%CI)
	No (N = 25,760)			Yes (N = 6440)				
	event	Person years	rate	event	Person years	rate		
Sex								
Male	111	82,421	1.35	52	20,957	2.48	1.85 (1.33, 2.57)***	1.66 (1.19, 2.32)**
Female	99	88,437	1.12	50	22,295	2.24	2.01 (1.43, 2.82)***	1.85 (1.31, 2.62)***
Age, years								
≤ 49	11	100,196	0.11	14	25,575	0.55	4.99 (2.26, 11.0)***	4.70 (2.10, 10.5)***
50–64	50	44,307	1.13	21	11,120	0.89	1.68 (1.01, 2.80)*	1.32 (0.78, 2.21)
≥ 65	149	26,356	5.65	67	6557	10.2	1.80 (1.35, 2.40)***	1.69 (1.26,2.26)***
Comorbidity^‡^								
No	46	114,349	0.40	22	24,374	0.90	2.25 (1.35, 3.74)**	2.71 (1.63, 4.50)***
Yes	164	56,509	2.90	80	18,878	4.24	1.47 (1.12, 1.92)**	1.61 (1.23, 2.10)***

Rate per 1000 person-year; Crude HR, relative hazard ratio

^†^Model was adjusted for age and comorbidities of diabetes, hypertension, hyperlipidemia, stroke, depression, coronary artery disease, head injury, and chronic kidney disease and medication of NSAIDs

^‡^Patients with any one of the comorbidities (diabetes, hypertension, hyperlipidemia, stroke, depression, coronary artery disease, head injury, chronic kidney disease and epilepsy) were classified as the comorbidity group

^*^p < 0.05, **p < 0.01, ***p < 0.001

Table 4 demonstrates the incidence and aHR of PD stratified by medication in patients with AS. Compared with patients without AS, the AS patients receiving immunosuppressant therapy had a significant increase of risk of Parkinson's disease (aHR 2.40; 95% CI 1.26–4.56, *p* < 0.01), followed by those without biological therapy (aHR 1.75; 95% CI 1.38–2.23, *p* < 0.001) and those without immunosuppressant therapy (aHR 1.70; 95% CI 1.32–2.18, *p* < 0.001). In this AS cohort, 37 patients received anti-TNF-ɑ biological therapy and none developed PD.

**Table 4:** Incidence and adjusted hazard ratio of Parkinson's disease stratified by medication in patients with ankylosing spondylitis

Medication exposed	N	Event	PY	Rate	Crude HR (95% CI)	Adjusted HR^†^ (95% CI)
Without ankylosing spondylitis	25,760	210	170,858	1.23	1 (Reference)	1 (Reference)
Ankylosing spondylitis						
Without Immunosuppressant therapy	5158	92	34,682	2.65	2.16 (1.69, 2.76)***	1.70 (1.32, 2.18)***
With Immunosuppressant therapy	1282	10	8569	1.17	0.95 (0.50, 1.79)	2.40 (1.26, 4.56)**
Without Biological therapy	6403	102	43,029	2.37	1.93 (1.53, 2.45)***	1.75 (1.38, 2.23)***
With Biological therapy	37	0	223		–	–

Rate per 1000 person-year; Crude HR, relative hazard ratio

^†^ Model was adjusted for age and comorbidities of diabetes, hypertension, hyperlipidemia, stroke, depression, coronary artery disease, head injury, and chronic kidney disease and medication of NSAIDs

^*^p < 0.05, **p < 0.01, ***p < 0.001

## Discussions

In this study, we proposed AS as a risk factor for the development of PD. As per our knowledge, the association between AS and PD has rarely been investigated [17]. This was the first extensive analysis of a nationwide population database of patients with AS and PD. The result indicated that the AS patients had significantly more comorbidities, including diabetes, hypertension, hyperlipidemia, depression, coronary artery disease, head injury and chronic kidney disease than matched controls. More importantly, AS was associated with an increased risk of PD development. The crude HR of 1.92, with an aHR of 1.75, for PD development was higher in the patients with AS compared with non-AS patients. Moreover, higher risk of PD in AS cohort without comorbidity indicated AS as a potential independent risk factor for PD.

Our result agreed with a recent US claims-based study which revealed that patients with AS were shown to have significantly more comorbidities, such as hypertension, coronary artery disease, depression and dyslipidemia than matched controls. Further follow-up in that study demonstrated a higher proportion of patients developed newly diagnosed cases of hypertension, coronary artery disease, depression, osteoporosis, spinal fracture, inflammatory bowel disease, psoriasis, and uveitis than matched controls after AS diagnosis [21].

The hypothesis that chronic inflammatory autoimmune diseases have an impact on the development of PD has been examined in several studies. Nationwide population-based researches from Taiwan and Sweden showed increased risk of PD among patients with autoimmune diseases [16, 17]. While in another study from Denmark, no overall association was found [22]. More importantly, recent genome-wide association studies (GWAS) and pathway analyses facilitated the identification of genetic overlap, i.e. pleiotropic loci, between PD and some autoimmune diseases. The results suggested that the PD-associated loci may contribute to PD through immune defects, supporting the presence of interaction between the immune system and neurodegeneration in PD [23]. Further analyses are warranted to figure out the possible pleiotropy between AS and PD.

Given that PD risk was higher in the patients with AS, our next question was whether the increase of PD risk was due to AS treatment. To address the effect of AS treatment on the risk of PD, the incidence rate and aHR of PD stratified by medication in patients with AS were analyzed. Medical treatment of AS mainly consists of NSAIDs, immunosuppressants, biological agents including anti-TNF-α and IL-17 inhibitors. The goals of AS treatment include alleviation of pain, improvement of physical function and delay of structural damage [24–26]. In this study, NSAID use was more prevalent in the AS cohort than in the non-AS cohort. Among 6440 AS patients, 1282 (19.9%) received immunosuppressants, while 37 (0.57%) underwent biological therapy.

Although some previous researches showed controversial results about the association between NSAID use and the risk of PD [27, 28], our result revealed that the hazard ratio for PD was significantly lower in patients taking NSAIDs compared with patients not taking (aHR 0.69). Consistent with our finding, a recent dose–response meta-analysis of NSAID use and risk of PD demonstrated that the use of non-aspirin NSAIDs was significantly associated with lower PD risk (RR:0.91; 95% CI, 0.84–0.99) [29].

After adjustment for sex, age, comorbidities, and medication of NSAIDs, the AS patients treated with and without immunosuppressants, including sulfasalazine and methotrexate that are prescribed for non-axial diseases, both had higher risks of PD (aHR, 2.40 and 1.70, respectively). A seemingly paradoxical harmful effect of immunosuppressant use on the risk of PD among AS patients was observed here. However, those needed immunosuppressant treatment usually had higher disease activity and peripheral arthritis. Therefore, the increase of PD risk could be associated with stronger inflammation of the disease rather than immunosuppressant treatment. This assumption is supported by a population-based case–control study in the US examining the risk of PD in relation to use of immunosuppressants. The results suggested that sulfasalazine and methotrexate were neither contributory to nor clearly inversely associated with PD risk [30].

TNF-α is recognized as an important inflammatory mediator in AS and anti-TNF-α therapy has been applied for advanced disease. We observed that AS patients who did not receive anti-TNF-α biological agents had a higher risk (aHR, 1.75), while thirty-seven patients who received anti-TNF-α biological therapy did not develop PD. This possible protective effect of anti-TNF-α treatment on PD development was also observed in a RA cohort. [31].

In contrast to AS, previous studies showed reduced risk of PD in patients with RA and SLE [22, 31, 32]. The aHR for PD development was 0.65 (95% CI, 0.58–0.73) in the RA cohort relative to the non-RA cohort [31]. On the other hand, an inverse association between SLE and the risk of subsequent PD, with aHR being 0.68 (95% CI, 0.51–0.90), was found [32]. Our observation of AS being associated with increased risk of PD seemed contradictory, under the premise that RA and SLE both correlated inversely with risk of PD development. However, the decreased risk observed among patients with RA and SLE could be explained by protective effect of the treatment with aggressive immune-suppressive drugs over prolonged periods [31, 32]. For example, inosine monophosphate dehydrogenase inhibitors and corticosteroids, both of which are frequently used in RA and SLE but not AS, were both associated with a lower risk of PD [30]. Further research is needed to disclose the relationship and underlying mechanism between AS and PD. Prospective studies are required to determine whether specific immunosuppressants are beneficial in preventing PD in autoimmune diseases.

There are some potential limitations in this study. First, the database does not provide complete information on clinical, laboratory, and radiographical examinations. The AS disease activity could not be tracked. Neither could we obtain information on a family history of PD and risk factors for PD such as smoking habits, alcohol consumption, occupation, and lifestyle [33–36]. We were unable to depict further possible pathogenesis of PD in patients with AS. Second, detailed prescription records of medication including doses, timing, and durations are unavailable. Third, whether the medical instruction was fully complied by outpatients with AS cannot be confirmed. Fourth, statistical quality of a retrospective cohort study is generally lower than those from randomized trials owing to possible biases related to adjustments for confounding variables.

## Conclusion

The observational studies would be leading the clinical reference value as real-world evidence and estimated the underlying risk for PD, thus, we demonstrated that the patients with AS existed an elevated risk of subsequent PD. However, the prospective study design would be still required to assess whether different immune modulators existed benefits to PD risk in patients with autoimmune diseases. The underlying mechanism and causality need to be further investigated and clarified.

## Data Availability

All the data is underlying the present study from the National Health Insurance Research database (NHIRD) and Management Office for Health Data, China Medical University Hospital, Taichung, Taiwan. Local interested researchers or cooperators can obtain the data through formal application to the Ministry of Health and Welfare, Taiwan.

## Abbreviations

CI: Confidence interval
aHR: Adjusted hazard ratio
ICD-9-CM: International Classification of Diseases, Ninth Revision, Clinical Modification
IRR: Incidence rate ratio
NHI: Taiwan’s National Health Insurance
NHIRD: National Health Insurance Research Database
AS: Ankylosing spondylitis
PD: Parkinson’s disease
